# Description of a CSF-Enriched miRNA Panel for the Study of Neurological Diseases

**DOI:** 10.3390/life11070594

**Published:** 2021-06-22

**Authors:** María Muñoz-San Martín, Imma Gomez, Albert Miguela, Olga Belchí, René Robles-Cedeño, Ester Quintana, Lluís Ramió-Torrentà

**Affiliations:** Neuroimmunology and Multiple Sclerosis Unit (UNIEM), Girona Biomedical Research Institute (IDIBGI), Doctor Josep Trueta University Hospital, Dr Castany s/n, Salt, 17190 Girona, Spain; mmunoz@idibgi.org (M.M.-S.M.); igomez@idibgi.org (I.G.); amiguela@idibgi.org (A.M.); lgabegui@gmail.com (O.B.); rrobles@idibgi.org (R.R.-C.)

**Keywords:** CSF, miRNAs, neurological diseases, OpenArray

## Abstract

Background: The study of circulating miRNAs in CSF has gained tremendous attention during the last years, as these molecules might be promising candidates to be used as biomarkers and provide new insights into the disease pathology of neurological disorders. Objective: The main aim of this study was to describe an OpenArray panel of CSF-enriched miRNAs to offer a suitable tool to identify and characterize new molecular signatures in different neurological diseases. Methods: Two hundred and fifteen human miRNAs were selected to be included in the panel, and their expression and abundance in CSF samples were analyzed. In addition, their stability was studied in order to propose suitable endogenous controls for CSF miRNA studies. Results: miR-143-3p and miR-23a-3p were detected in all CSF samples, while another 80 miRNAs were detected in at least 70% of samples. miR-770-5p was the most abundant miRNA in CSF, presenting the lowest mean Cq value. In addition, miR-26b-5p, miR-335-5p and miR-92b-3p were the most stable miRNAs and could be suitable endogenous normalizers for CSF miRNA studies. Conclusions: These OpenArray plates might be a suitable and efficient tool to identify and characterize new molecular signatures in different neurological diseases and would improve the yield of miRNA detection in CSF.

## 1. Introduction

Neurological disorders are diseases that might affect the peripheral and central nervous system (CNS), affecting hundreds of millions of individuals of all age groups and races worldwide [1,2]. Neurological disorders were responsible for 276 million disability-adjusted life-years and 16.5% of total global deaths in 2016 [3]. Specifically, brain disorders, which include a broad range of different conditions, suchas neurodegenerative diseases, demyelinating and neuroinflammatory diseases, tumors, dementias, infections or mental disorders, are a major public health problem, representing an important economic and social burden [4]. The brain is considered to be the most complex organ of the body, but its inaccessibility hinders the study of pathological processes [5].

Cerebrospinal fluid (CSF) is an ultrafiltrate of plasma that is found around and within the organs of the CNS and maintains an appropriate chemical environment for the neural tissue [6]. In different neurological disorders, the composition of CSF might change [7], highlighting its interesting nature as a fluid, as it might reflect the level of brain damage [8]. Measuring levels of different components of CSF might be a valuable tool to facilitatethe diagnosis and prognosis of neurological conditions [7].

microRNAs (miRNAs) are small non-coding RNAs that are evolutionarily conserved whose mature and biologically active form is 18–25 nucleotides long [9]. miRNAs regulate gene expression by two mutually exclusive posttranscriptional mechanisms: translational repression or mRNA cleavage [10]. Most miRNAs are located inside cells, but there are also extracellular miRNAs, known as circulating miRNAs, that might be found in different biological fluids such as plasma, serum or CSF [11]. In contrast to cellular miRNAs, circulating miRNAs are remarkably stable despite the existence of RNases in body fluids and unfavorable conditions [12]. It has been suggested that they are delivered to the extracellular fluids by the passive leakage of apoptosis, necrosis or due to active secretion by cells [13]. In 2007, Valadi et al. proposed a novel paracrine mechanism for intercellular communication showing that extracellular miRNAs could be delivered into recipient cells, where they could alter gene expression [14].

CSF circulating miRNA studies in neurological conditions have gained tremendous attention during the last years, as they might be promising candidates to be used as biomarkers and provide new insights into the disease pathology and therapeutic targets of neurological disorders. CSF miRNAs might discriminate between Alzheimer’s disease (AD) individuals and controls [15] or distinguish multiple sclerosis (MS) disease phenotypes from each other [16]. The diagnosis of other neurological conditions such as Huntington’s disease (HD), temporal lobe epilepsy or CNS injury might be supported by the analysis of CSF miRNAs [17,18,19]. It is known that CSF miRNA content is less abundant than the content of other biological material such as cells or serum [20], and miRNA composition can vary between tissues and biofluids [21].

miRNA profiling studies show great promise for the biomarker field in neurological disorders, but further research is needed to validate theresults ofdifferent laboratories [22]. Therefore, the main aim of this study was to describe a panel of CSF-enriched miRNAs that might be a suitable and efficient tool to identify and characterize new molecular signatures in different neurological diseases and improve the yield of miRNA detection in CSF. A set of 215 miRNAs was selected to be included in the customized panels, and their expression and abundance in CSF samples were analyzed. In addition, some miRNAs were proposed as suitable endogenous controls for CSF miRNA studies.

## 2. Material and Methods

### 2.1. Biological Samples

The whole cohort of patients was composed ofspinal anesthesia subjects (SAS), corresponding to neurologically healthy patients with hip/knee impairment undergoing surgical intervention; subjects affected by other neurological diseases (ONDs) ratherthan MS, whose pathologies were of vascular origin, migraines, dementia or dizziness; and MS subjects, including the phenotypes relapsing–remitting MS (RRMS) and primary progressive MS (PPMS). Most individuals were recruited at the Girona Neuroimmunology and Multipe Sclerosis Unit of Dr. Josep Trueta University Hospital (Girona, Spain). All participants signed a written informed consent form. The Ethics Committee and the Committee for Clinical Investigation from Dr. Josep Trueta University Hospital approved the protocol employed.

CSF samples were obtained at the moment of diagnosis by means of a lumbar puncture made by a neurologist. In the case of SAS, an anesthesiologist performed this technique during surgery. After collecting CSF, it was centrifuged at 400× *g* and 19 °C for 15 min in order to obtain cell-free CSF.

### 2.2. Circulating RNA Extraction and Purification

Circulating RNA from CSF was purified from 300 or 500 μL of starting material using the mirVana PARIS Isolation kit (Applied Biosystems) according to the manufacturer’s protocol. Briefly, the initial volume of sample (300 μL in most cases) was mixed with the same volume of 2× Denaturing solution containing 375 μL of 2-mercaptoethanol. At this point, two exogenous miRNAs (cel-miR-39 and cel-miR-54) were added at 5 pM to verify the quality of the extraction process. The same volume of acid-phenol:chloroform was then added, and the upper aqueous phase obtained after centrifugation (17,000× *g*, 10 min, 19 °C) was recovered. This phase was mixed with 100% ethanol and placed into a filter cartridge provided in the kit. After the RNA washing procedures, total RNA was eluted with 40 µL of nuclease-free water and stored at −80 °C for its lateruse.

### 2.3. Circulating miRNA Retrotranscription and Preamplification

The Applied BiosystemsTaqMan Advanced miRNA cDNA Synthesis kit (Applied Biosystems, Foster City, CA, USA) was used to obtain miRNA cDNA in this study. This kit has been specially designed to work with materials whose miRNA contents are limited. It employs universal reverse transcription (RT) chemistry to obtain the cDNA template used for mature miRNA detection and quantification with TaqMan Advanced miRNA Assays. Two microliters of RNA eluate were used for the preparation of miRNA cDNA and, after the addition of a poly(A) tail and an adaptor following the manufacturer’s instructions, an RT reaction and miRNA amplification reaction were performed.

### 2.4. Circulating miRNA Profiling

To analyze the miRNA expression ofCSF samples, cDNA templates were subjected to PCR amplification to detect specific miRNAs using TaqMan Advanced miRNA assay technology. These assays were pre-loaded during the manufacturing process in TaqMan OpenArray Human Advanced microRNA plates—the high-throughput screening platforms chosen to carry out the profiling step in this study. Two different formats were used: fixed-content plates (fc-OA) and custom-configured plates (cc-OA). The first werepre-loaded with 754 human Advanced miRNA assays and allowed the analysis of three samples per plate. cc-OA plates were designed to analyze specifically 215 CSF-enriched miRNAs for this study (Supplementary Table S1).

cDNA templates were diluted to 1:20 in 0.1X TE buffer to be run in triplicate in TaqMan OpenArray Plates. These diluted cDNA samples were combined with the same volume of TaqMan OpenArray Real-Time PCR Master Mix in tubes. Five microliters of the combined master mix and cDNA sample were added to the determined wells in an OpenArray 384-well Sample Plate. The automated OpenArray AccuFill System was used to load the samples into the TaqMan OpenArray Plate through holes. Then, they were cycled and imaged with the QuantStudio 12 K Flex Real-Time PCR System, resulting in Cq values for each sample and miRNA.

### 2.5. Databases for Cellular/Tissue-Enriched Source Analyses and Disease Associations

To study the potential sources of the 20 most abundant miRNAs in CSF, the human miRNA tissue atlas, CNS microRNA profiles described by Hoye et al. and FANTOM5 human miRNA atlas were used to identify miRNA expression across tissue, CNS cells and primary cells, respectively [21,23,24]. Version 3.0 of the Human MicroRNA Disease Database was used to explore experimentally supported miRNA–disease associations [25].

### 2.6. Search of Candidate Normalizer miRNAs for CSF Samples

Three different algorithms were used to identify stable miRNAs in CSF samples: Normfinder [26], geNorm [27] and the coefficient of variation (CV) score. These algorithms generate a score that represents the stability: the smaller the score, the higher the expression stability the miRNA has. The summarized stability score (SSS) for each miRNA was calculated to summarize the results [28].

## 3. Results

### 3.1. Profiling of CSF Samples in fc-OA Plates

Three samples of CSF were used to extract RNA from an initial volume of 500 μL and 300 μL in order to establish the average miRNA detection in CSF samples using two fc-OA panels containing 754 TaqMan Advanced miRNA assays.

**Figure 1:**
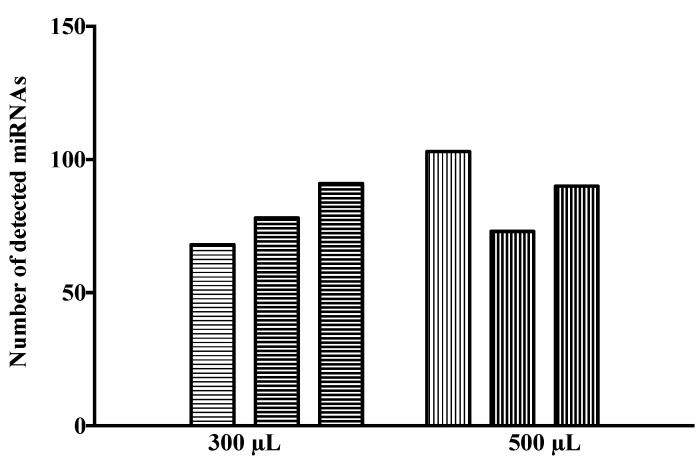
miRNA detection in CSF in TaqMan OpenArray Human Advanced microRNA panels. The number of miRNAs with Cq values (range 15–35) detected in each tested sample was represented in each column. Columns with horizontal stripes represent CSF samples whose initial volume was 300 μL and columns with vertical stripes represent CSF samples whose initial volume was 500 μL.

As observed in Figure 1, 500 μL of CSF samples presented an average number of detected miRNAs of 89, whereas 300 μL of CSF samples showed a mean detection of 79 miRNAs, which indicated a percentage of detection of 11.8% and 10.5%, respectively, from the total miRNA set. The detection of 99 miRNAs overlapped for at least one sample from each starting volume. Analyzing each initial volume individually, 23, 60 and 112 miRNAs were detected in 3, 2 or 1 sample/s for 300 μL of CSF, respectively, corresponding to 13.4%, 21.5% and 65.1% of the total set of detected miRNAs. Regarding to 500 μL, 52, 39 and 59 miRNAs were detected in 3, 2 or 1 sample/s, respectively, corresponding to 34.7%, 26.0% and 39.3% of the total detected miRNAs (Supplementary Figure S1). Despite the fact that both initial volumes presented a comparable detection, the intra-volume degree of correlation when working with 300 μL of CSF was less consistent than that observed for 500 μL of CSF. However, as CSF is very valuable and difficult to obtain, henceforth, total RNA from CSF was extracted from 300 μL of sample.

### 3.2. Selection of 215 miRNAs to Be Included in cc-OA Plates

Due to the low detection presented by CSF samples in fc-OA, cc-OA plates were customized for studying CSF miRNA profiles. The selected 224 format allowed us to analyze 12 samples simultaneously, covering a total of 215 miRNAs plus one mandatory control (miR-16). miRNAs needed to meet at least one of the following criteria to be included in the panel:

1. Previously associated with MS in tissue, serum/plasma or CSF;
2. Particularly brain-enriched;
3. Detectable in CSF based on existing literature and/or our previous experience;
4. Potential endogenous normalizer;
5. Negative control.

The list of 215 miRNA assays included in these cc-OA panels is shown in Supplementary Table S1.

### 3.3. miRNA Classification According to Their Detectability

To analyze the performance of the detection of miRNAs with these cc-OA plates, 64 CSF samples were used, belonging to SAS, OND, PPMS and RRMS individuals. The clinical characteristics of the cohort are depicted in Table 1.

miRNAs were classified according to the percentage of CSF samples in which they were detectable (Table 2). From the total set of 215 miRNAs, 2 miRNAs, miR-143-3p and miR-23a-3p, were detected in all CSF samples. Another 80 miRNAs, representing 37.2% of miRNAs, were detected in at least 70% of samples. These two categories represented the suitable miRNAs to proceed with further analysis in differential expression studies. When subclassifying the whole cohort in each specific individual group, it could be observed that 80 miRNAs might be detectable in 70% of samples in at least three groups. Other miRNAs might seem to be more detectable in some groups exclusively (Supplementary Figure S2).

### 3.4. miRNA Abundance in CSF Samples and Disease Associations

Among those miRNAs that were detectable in at least 70% of samples, the 20 most abundant miRNAs (lower Cq values) in CSF were identified by ranking the average Cq values of all samples as shown in Table 3. miR-770-5p presented the lowest mean Cq value (20.9). When subclassifying the whole cohort in each specific patient group, miR-770-5p remained the most abundant miRNA in CSF. From these 20 miRNAs, it should be highlighted that miR-451a and miR-144-3p presented the greatest differences between OND and SAS individuals.

Exploring the association of these miRNAs with diseases in the Human MicroRNA Disease Database [25], all of them have been previously related to at least one neurological disorder such as glioblastoma, Parkinson’s disease, MS, Alzheimer’s disease, stroke or epilepsy.

### 3.5. Cellular/Tissue-Enriched Source Analysis of Most Abundant miRNAs

**Figure 2:**
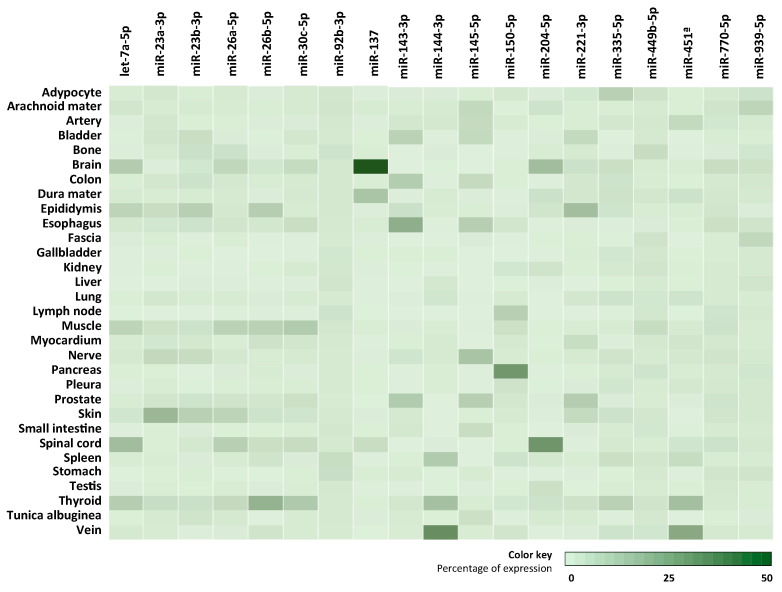
Most abundant miRNA expression in tissue biopsies by human miRNA tissue atlas. Normalized expression of the most abundant miRNAs in CSF in 30 tissues was retrieved from the human miRNA tissue atlas [20] and the percentage of expression calculated and represented in a heat map.

The potential tissue and cellular origin of the 20 most abundant miRNAs in CSF was examined using different repositories. First, Ludwig et al. determined the abundance of miRNA in tissue biopsies of two individuals [21]. Using the data from this human miRNA tissue atlas, a heat map of the normalized expression of miRNA in 30 different tissues was constructed (Figure 2). As could be observed, the expression of some miRNAs (let-7a-5p, miR-137, miR-204-5p, miR-221-3p, miR-26a-5p, miR-26b-5p, miR-30c-5p, miR-335-5p, miR-451a, miR-770-5p and miR-939-5p) was higher in brain and spinal cord tissues. Further investigating CNS cell types using the repository of CNS microRNA profiles [24], miR-137, miR-335-5p and miR-770-5p might present an increased expression in motor neurons, whereas miR-221-3p expression might seem to be elevated in astrocytes.

After analyzing miRNA expression across primary cells using the FANTOM5 atlas [23], the predominant expression of miR-26a-5p, miR-26b-5p, miR-144-3p, miR-150-5p and miR-450b-3p in different immune cell subsets should be highlighted (Table 4). Specifically, miR-143-3p was highly expressed in circulating cells and neutrophils, miR-150-5p was highly expressed in T cells and circulating cells, and miR-450b-3p was highly expressed in neutrophils. miR-26a-5p and miR-26b-5p were similarly found in the nine immune cells described.

### 3.6. Search for Suitable Endogenous Normalizers for CSF Samples

Although a normalization method based on the mean expression value of all miRNAs has been proposed and validated for qPCR data from array-based approaches (screening phase), the search fora candidate reference endogenous miRNAs is necessary for futurestudies, and thus we evaluated a limited panel of miRNAs in a wider cohort (validation phase) [28]. To date, no study has proposedreliable endogenous controls for CSF using TaqMan Advanced miRNA assay technology. For this reason, a search of endogenous controls for use in qPCR experiments with CSF samples was made using the OpenArray data obtained in this study.

Those miRNAs detected in at least 70% of samples were selected. Three different algorithms were used to identify stable miRNAs: Normfinder [26], geNorm [27] and the CV score. To outline the results, the SSS for each miRNA was also calculated [28]. In Table 5, stability scores obtained for the 17 most stable miRNAs are represented, showing that miR-23a-3p, miR-26b-5p and miR-125a-5p might be suitable endogenous miRNAs for CSF studies. SSS scores were also calculated for these 17 miRNAs in each group of patients (Supplementary Table S2). While miR-26b-5p presented similar scores in each group, miR-23a-3p seemed more stable in PPMS and SAS individuals and miR-125a-5p might not be recommendable for OND. miR-335-5p and miR-92b-3p could also be stable in all groups.

**Figure 3:**
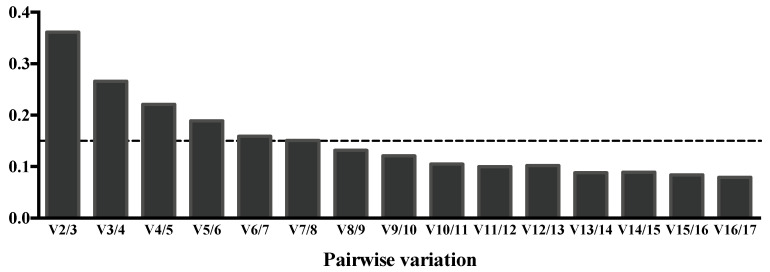
Evaluation of the optimum number of reference miRNAs for CSF samples according to the geNorm software. Pairwise variation between samples is reduced by the inclusion of additional reference miRNAs. The magnitude of the change in the normalization factor after the inclusion of a ninth additional reference gene implies a value under the recommended cut off of 0.15, showing that the use of eight endogenous controls is optimum for CSF samples.

Despite selecting the best endogenous candidates for CSF samples, the establishment ofthe optimum number of reference miRNAs must be experimentally determined. geNorm also generates a pairwise stability measure to determine if adding more reference miRNAs for the normalization process is beneficial. As shown in Figure 3, the recommended cut off value of 0.15 indicates that the use of eight endogenous controls in CSF samples would offer an acceptable stability for the reference miRNA combination [29]. Therefore, we would strongly recommend the use of miR-21-5p, miR-23a-3p, miR-26b-5p, miR-27a-3p, miR-92b-3p, miR-125a-5p, miR-221-3p and miR-335-5p for the normalization of qPCR experiments in CSF samples.

## 4. Discussion

CSF is a clear liquid located around and within the CNS, and it maybe analyzed through lumbar puncture [30]. One of its essential functions is the maintenance of an appropriate chemical environment for neural tissue. As the interstitial fluid of the CNS and CSF are in anatomic continuity, this valuable biofluid might mirror the events of the CNS [6]. Even though CSFis a sample that is obtained with a very invasive technique, it might be very useful for the study of the pathogenic mechanisms of neurological diseases as it is a relatively cell and microorganism-free fluid [31].

miRNAs have been detected in different biological fluids as plasma, serum or CSF, where they remain highly stable, unlike the case of cellular miRNAs [32]. For this reason, circulating miRNA profiles have been widely studied in different conditions to exploit their potential as biomarkers. miRNAs have been described to be implicated in different processes such as inflammation, neurogenesis, apoptosis, blood–brain barrier protection and/or remyelination [33,34]. The deregulation of their levels in patients with neurological diseases might represent new potential biomarkers as well as new avenues for research in developing new therapies [33,34,35].

Some of the unsolved challenges in miRNA profiling studies include the existence of heterogeneous and conflicting results as well as the lack of replication among studies [22]. In fact, as shown in this study, differences in the consistency of results might be observed depending on the initial volume of CSF. As CSF has an important role in diagnosis but its collection might be limited due to the invasiveness of this procedure [31], the use of 300 μL of CSF might still be encouraged, as the detectability was comparable to that observed with 500 μL of CSF, despite presenting a lower intra-volume correlation. High-throughput platforms allow the detection of multiple miRNAs in parallel, which is very useful in biomarker research in order to find molecular signatures [28]. Most published CSF profiling studies have used pre-configured miRNA detection platforms. These platforms have been frequently tested in biological fluids other thanCSF. miRNAs might present different levels of expression and their composition can vary between tissues and biofluids [21], and CSF contains lower levels of miRNA than serum or plasma [20]. It is necessary to analyze and study CSF miRNA profiles to design specific platforms that allow us to extract all the informative potential that CSF could offer more efficiently.

This study presents the first analysis of CSF miRNA levels using TaqMan Advanced miRNA technology from Applied Biosystems with the final aim of designing CSF-enriched miRNA panels to be used in a wide spectrum of neurological diseases. In 2017, Wang et al. defined a specific CSF–miRNA panel to be used in the study of AD [36]. They customized TaqMan low density array (TLDAs) panels containing 47 miRNAs. However, as was explained in Section 3, up to 79 miRNAs were detected in our studied cohort using fc-OA plates. Therefore, to customize OpenArray plates targeting 215 miRNAs, the new TaqMan Advanced miRNA technology was chosen. Among these 215 miRNAs, 41 were present in the panel of Wang et al. [36]. In addition, the utilization of OpenArray instead of TLDAs brings the efficient advantage of analyzing a larger number of samples in a shorter period of time [37], and this new TaqMan Advanced miRNA technology allows a more universal and specific detection of miRNA [38].

The performance of cc-OA plates was tested in order to determine their suitability to be used as a tool to identify new molecular signatures in CSF. First of all, the detectability of each assayed miRNA was measured by calculating the percentage of samples in which it could be detected. This analysis showed that miR-143-3p and miR-23a-3p were present in all samples, while another 80 miRNAs could be detected in at least 70% of samples, representing 38.1% of miRNAs. Second, the 20 most abundant miRNAs in the studied CSF samples were chosen by ranking their mean Cq value. All of them belonged to this 38.1% of miRNAs detected in 70% of samples. Surprisingly, only four of them (let-7a-5p, miR-30c-5p, miR-150-5p and miR-204-5p) were included in the CSF panel described by Wang et al. [36]. It should be mentioned that this discrepancy might be due to the use of different technologies for miRNA detection and the processing of CSF samples with different protocols in both studies. In addition, despite having been analyzed, only nine miRNAs were detected in previous miRNA studies carried out with human CSF samples and TLDAs [39,40]. This highlights the importance of using the newest technologies to increase specificity and sensibility. Although redefining the format by reducing the number of assays might be an option for future work, we would suggest that this format should be maintained to be able to find new miRNA profiles in different neurological disorders, as we could only use a narrow range of available CSF samples.

When examining the potential source of the most abundant miRNAs in CSF, some of them were highly expressed in the brain, spinal cord and different CNS cell types. These miRNAs have been found to be associated with some of the most common CNS disorders in the contexts of tumors (glioblastoma) [41], neurodegenerative aspect (Parkinson’s disease) [42], dementia (AD) [43] or long-term disability (stroke) [44]. Another interesting group of miRNAs is those whose expression was found to be increased in specific immune cell subsets such as miR-26a-5p, miR-26b-5p, miR-144-3p, miR-150-5p and miR-450b-3p. Specifically, it should be highlighted that miR-150-5p has been previously related to MS in CSF [39,40], a chronic inflammatory and neurodegenerative disease of the CNS [45], and its key role in modulating inflammatory responses has been widely recognized [46].

An essential step in qPCR experiments is the normalization procedure, which enables the control of variations in extraction and RT yield, as well as increased efficiency of amplification. It is required before any comparison in miRNA concentrations between different samples and biological groups is performed [47]. The search for candidate endogenous miRNAs will be necessary in later studies [28]. In this study, three different methods were in CSF samples. The NormFinder approach calculated the stability based on the intergroup and intragroup variation, the GeNorm algorithm ranked genes based on pairwise correlation, and CV analysis calculated the variance of a miRNA across all samples taken together [48]. In addition, SSS, as proposed by Marabita et al., was calculated in order to summarize all this information [28].

miR-23a-3p, miR-26b-5p and miR-125a-5p were found to be the most stable miRNAs in the whole cohort of CSF samples. However, when analyzing the stability in the different groups of individuals, miR-26b-5p, miR-92b-3p and miR-335-5p might seem the most promising miRNAs to be used as endogenous normalizers. This reinforces the necessity of the experimental validation of any endogenous miRNAs as normalizers for particular tissues, cell types or biofluids and specific experimental designs [49]. Although miR-23a-3p might have been proposed as an optimal reference miRNA in cervical tissue [50], it has also been found to be involved in some aspects related to melanoma growth and progression [51]. Some other miRNAs previously used as normalizers in CSF in other studies are miR-24 [52], miR-17 [39] and miR-320a [53]. Although the preferred method for the normalization of individual qPCR data is the utilization of a minimum of two endogenous reference miRNAs [54], our analysis determined that eight endogenous miRNAs is the optimum number for CSF samples, with the addition of miR-21-5p, miR-27a-3p and miR-221-3p to be combined with those previously mentioned.

## 5. Conclusions

As the interest in high-throughput platforms is increasing in the field of miRNA biomarkers, a panel of CSF-enriched miRNAs was presented and a set of endogenous controls to be used in neurological diseases was proposed.

These cc-OA plates with 215 loaded miRNA assays allowed the detection of approximately 38.1% of these miRNAs in at least 70% of CSF studied samples, withmiR-770-5p having the lowest Cq values. Although the use of eight endogenous controls in CSF samples is highly recommended, miR-26b-5p, miR-335-5p and miR-92b-3p are the most stable miRNAs in CSF.

These OpenArray plates might be a suitable and efficient tool to identify and characterize new molecular signatures in different neurological diseases and would improve the yield of miRNA detection in CSF.

## Figures and Tables

**Table 1:** Clinical characteristics of the studied cohort.

Group	*n*	Age (Mean ± SD)	Sex (F/M)
SAS	7	50.2 ± 5.8	5/2
OND	6	51.7 ± 5.5	4/2
Vascular origin	2	50.5 ± 0.7	2/0
Migraines	2	52 ± 5.7	2/0
Dementia	1	60	0/1
Dizziness	1	45	0/1
PPMS	11	52.6 ± 7.0	6/5
RRMS	40	32.1 ± 13.0	30/10

SAS: spinal anesthesia subjects; OND: other neurological diseases; PPMS: primary progressivemultiple sclerosis; RRMS: relapsing-remitting multiple sclerosis; SD: standard deviation; F: female; M: male.

**Table 2:** miRNA classification according to their detectability.

Detection	Number of miRNAs (%)	miRNAs
100%	2 (0.9)	miR-143-3p; miR-23a-3p
99–70%	80 (37.2)	let-7a-5p; let-7b-5p; let-7c-5p; let-7f-5p; let-7g-5p; let-7i-5p; miR-100-3p; miR-100-5p; miR-101-3p; miR-10b-5p; miR-124-3p; miR-125a-5p; miR-125b-5p; miR-1260a; miR-1298-5p; miR-130a-3p; miR-137; miR-142-3p; miR-144-3p; miR-145-5p; miR-146a-5p; miR-148a-3p; miR-148b-3p; miR-150-5p; miR-151a-3p; miR-15a-5p; miR-181a-5p; miR-181c-5p; miR-185-5p; miR-186-5p; miR-195-5p; miR-199a-3p; miR-199a-5p; miR-19a-3p; miR-204-5p; miR-20a-5p; miR-21-5p; miR-219a-5p; miR-22-3p; miR-221-3p; miR-223-3p; miR-23b-3p; miR-24-3p; miR-25-3p; miR-26a-5p; miR-26b-5p; miR-27a-3p; miR-27b-3p; miR-29a-3p; miR-29c-5p; miR-30c-5p; miR-30d-5p; miR-320a; miR-320b; miR-335-5p; miR-338-3p; miR-342-3p; miR-34a-5p; miR-34c-5p; miR-361-5p; miR-374b-5p; miR-376a-3p; miR-378a-3p; miR-423-5p; miR-448; miR-449b-5p; miR-450b-3p; miR-451a; miR-452-3p; miR-497-5p; miR-645; miR-652-3p; miR-653-3p; miR-660-5p; miR-664a-3p; miR-770-5p; miR-885-5p; miR-9-5p; miR-92b-3p; miR-939-5p
69–50%	31 (14.4)	let-7b-3p; let-7e-5p; miR-1-3p; miR-103a-3p; miR-107; miR-128-3p; miR-133a-3p; miR-133b; miR-135a-5p; miR-151a-5p; miR-15b-5p; miR-17-5p; miR-1911-5p; miR-193a-5p; miR-196a-5p; miR-222-3p; miR-28-5p; miR-30c-2-3p; miR-34b-3p; miR-34b-5p; miR-34c-3p; miR-378a-5p; miR-424-5p; miR-501-3p; miR-516b-5p; miR-525-3p; miR-633; miR-9-3p; miR-93-5p; miR-99a-3p; miR-99b-5p
49–30%	31 (14.4)	let-7f-2-3p; miR-106b-3p; miR-106b-5p; miR-126-5p; miR-1264; miR-132-3p; miR-155-5p; miR-181b-5p; miR-190a-5p; miR-205-5p; miR-210-3p; miR-302b-3p; miR-302d-3p; miR-31-5p; miR-32-5p; miR-339-5p; miR-361-3p; miR-376c-3p; miR-411-5p; miR-412-3p; miR-425-5p; miR-483-3p; miR-484; miR-502-3p; miR-505-3p; miR-518f-3p; miR-524-3p; miR-576-3p; miR-583; miR-92a-3p; miR-937-3p
30–1%	70 (32.6)	miR-103a-2-5p; miR-10a-5p; miR-122-5p; miR-1247-5p; miR-1249-3p; miR-125a-3p; miR-127-3p; miR-129-2-3p; miR-1292-5p; miR-142-5p; miR-145-3p; miR-146b-5p; miR-153-3p; miR-181d-5p; miR-183-3p; miR-191-3p; miR-191-5p; miR-194-5p; miR-19b-3p; miR-200c-3p; miR-203a-3p; miR-206; miR-216a-5p; miR-218-5p; miR-27b-5p; miR-30a-3p; miR-30c-1-3p; miR-30e-3p; miR-323a-3p; miR-325; miR-326; miR-328-3p; miR-34a-3p; miR-363-3p; miR-369-3p; miR-369-5p; miR-373-3p; miR-375; miR-383-5p; miR-410-3p; miR-449a; miR-450b-5p; miR-452-5p; miR-454-3p; miR-455-3p; miR-483-5p; miR-486-5p; miR-487a-3p; miR-489-3p; miR-490-3p; miR-513a-5p; miR-515-3p; miR-518d-3p; miR-518e-3p; miR-520h; miR-523-3p; miR-532-3p; miR-532-5p; miR-548d-5p; miR-548e-3p; miR-548k; miR-548n; miR-551a; miR-570-3p; miR-593-5p; miR-615-3p; miR-628-3p; miR-642a-5p; miR-656-3p; miR-876-3p
0%	1 (0.5)	miR-211-5p

**Table 3:** miRNA abundance in CSF samples.

miRNA	Mean Cqvalue	Neurologicaldisease	PMID
miR-770-5p	20.9	GBM	27572852
miR-939-5p	24.1	Complex regional painsyndrome	31489147
miR-450b-3p	24.1	PD	23938262
miR-26b-5p	24.4	AD, hypoxia/ischemia, diffuseintrinsicpontine glioma, ALS	23895045, 29937716, 0124166, 29543360, 30210287, 24027266
miR-145-5p	24.7	Myastheniagravis, MS, stroke, seizure, GBM	24043548, 23773985, 26096228, 27833019, 28284220, 23745809, 27752929,
miR-204-5p	25.0	Frontotemporaldementia, SPI, mesial temporal lobeepilepsy, GBM	29434051, 29547407, 25410734, 30008822
miR-30c-5p	25.1	ALS, MS	30210287, 29551498
miR-451a	25.1	Depression, ALS, GBM	26343596, 30210287, 18765229
miR-335-5p	25.4	Stroke, astrocytoma, neuroblastoma, majordepressiondisorder	27856935, 21592405, 23806264, 26314506
let-7a-5p	25.8	PD, GBM, ALS, MS	30267378, 23600457, 26502847, 30210287, 25487315
miR-23a-3p	25.9	MS, epilepsy, HD, SPI, GBM	24277735, 26382856, 30359470, 27725128, 27907012, 20711171
miR-221-3p	26.0	Stroke, PD, GBM, neuropathicpain	23860376, 27748571, 28381184, 27059231, 18759060, 24055409
miR-449b-5p	26.0	Stroke, PD	30135469, 29935433
miR-144-3p	26.0	Bipolar disorder, GBM, AD	19849891, 26250785, 23546882
miR-143-3p	26.2	AD, GBM	26078483, 22490015, 23376635, 21211035
miR-137	26.2	AD, schizophrenia, GBM, HD	22155483, 26899870, 29684772, 26187071, 21926974, 25044277, 18577219, 23965969, 21994399
miR-150-5p	26.4	MS, stroke, HD	28067602, 27144214, 27246008, 22048026
miR-26a-5p	26.5	Migraine, PD, GBM	26333279, 30267378, 20080666
miR-92b-3p	27.3	Neuroblastoma, GBM	21572098, 22829753
miR-23b-3p	27.4	GBM	22745829, 23152062

Disease association established using the Human MicroRNA Disease Database [24]. PMID numbers identify articles referring an association between a miRNA and a neurological disease. GBM: glioblastoma; PD: Parkinson’sdisease; AD: Alzheimer’s disease; ALS: amyotrophic lateral sclerosis; MS: multiple sclerosis; SPI: spinal cord injury; HD: Huntington’s disease.

**Table 4:** Percentage of expression of the most abundant miRNAs in primary immune cells.

Immune Cell Subset	miR-26a-5p	miR-26b-5p	miR-144-3p	miR-150-5p	miR-450b-3p
Circulating cell	4.87	6.47	53.67	25.63	0.00
Dendritic cell	1.47	3.03	0.65	0.71	0.00
Lymphocyte B lineage	4.77	6.71	1.76	3.35	0.00
Macrophage	1.69	2.84	0.00	0.03	0.00
Mastcell	3.41	7.97	0.60	0.08	0.00
Monocyte	3.58	5.75	0.90	5.08	0.00
Natural Killer cell	3.65	7.26	0.24	17.09	0.00
Neutrophil	5.91	8.69	23.93	0.28	27.95
T cell	4.25	4.85	0.23	46.94	0.00

Data obtained from FANTOM5 human miRNAs repository [22]. Percentage of expression in specific primary cells are shown for each miRNA.

**Table 5:** miRNA stability scores for geNorm, Normfinder and CV algorithms and SSS score for CSF samples.

miRNA	geNorm	NormFinder	CV Score	SSS Score
miR-101-3p	1.63 (12)	0.83 (12)	0.98 (17)	2.07 (14)
miR-125a-5p	1.46 (4)	0.63 (3)	0.59 (3)	1.69 (3)
miR-143-3p	1.59 (8)	0.79 (8)	0.73 (9)	1.92 (9)
miR-151a-3p	1.71 (15)	0.90 (15)	0.85 (12)	2.11 (15)
miR-15a-5p	1.71 (16)	0.91 (16)	0.93 (16)	2.15 (17)
miR-181a-5p	1.67 (16)	0.87 (14)	0.80 (11)	2.05 (13)
miR-186-5p	1.72 (17)	0.92 (17)	0.88 (13)	2.14 (16)
miR-21-5p	1.66 (6)	0.74 (6)	0.62 (5)	1.82 (6)
miR-221-3p	1.61 (11)	0.81 (11)	0.61 (4)	1.90 (8)
miR-23a-3p	1.43 (1)	0.61 (1)	0.65 (6)	1.68 (2)
miR-26b-5p	1.46 (3)	0.64 (4)	0.46 (1)	1.66 (1)
miR-27a-3p	1.58 (7)	0.77 (7)	0.71 (8)	1.89 (7)
miR-335-5p	1.52 (5)	0.72 (5)	0.56 (2)	1.77 (5)
miR-652-3p	1.60 (10)	0.79 (9)	0.91 (15)	2.00 (12)
miR-653-3p	1.60 (9)	0.80 (10)	0.89 (14)	1.99 (11)
miR-9-5p	1.64 (13)	0.83 (13)	0.76 (10)	1.99 (10)
miR-92b-3p	1.44 (2)	0.62 (2)	0.66 (7)	1.70 (4)

miRNA stability scores are represented for each algorithm and its ranked position from the total set of 17 miRNAs is in brackets. CV: coefficient of variation; SSS: summarized stability score.

## Data Availability

The data presented in this study are available on request from the corresponding author.

## Supplementary Materials

The following are available online at https://www.mdpi.com/article/10.3390/life11070594/s1. Table S1: List of selected miRNA assays included in TaqMan OpenArray Human Advanced microRNA panels, Table S2: SSS scores for CSF samples of each studied group, Figure S1: Venn diagram plot the number of detected miRNAs in different conditions, Figure S2: Venn diagram plot the number of miRNAs detected in at least 70% of samples in each group of patients, Figure S3: Mean Cq value of the most abundant miRNAs in CSF in each specific group of studied samples.
