# Supplementary material for: Description of a CSF-Enriched miRNA Panel for the Study of Neurological Diseases
Source: Life (Basel). 2021 Jun 22;11(7):594. doi: 10.3390/life11070594 (PMC8305419; doi:10.3390/life11070594)
Supplement: Supplementary file 1 [file life-11-00594-s001.zip › life-1247829-supplementary.pdf]

life

MDPI

Supplementary material of Description of a CSF-Enriched miRNA Panel for the Study of Neurological Diseases

Table S1. List of selected miRNA assays included in TaqMan OpenArray Human Advanced microRNA panels.

miRNA	Assay Code	MS-Associated miRNA	Brain-Enriched miRNA	CSF-Detectable miRNA	Potential Normalizer	Negative Control
ath-miR-159a	478411					X
let-7a-5p	478575	X		X		
let-7b-3p	478221			X		
let-7b-5p	478576			X		
let-7c-5p	478577		X	X		
let-7e-5p	478579	X		X		
let-7f-2-3p	477843			X		
let-7f-5p	478578	X		X		
let-7g-5p	478580	X	X	X		
let-7i-5p	478375			X		
miR-1-3p	477820	X		X		
miR-9-5p	478214		X	X		
miR-9-3p	478211			X		
miR-10a-5p	479241			X		
miR-10b-5p	478494			X		
miR-15a-5p	477858	X		X		
miR-15b-5p	478313	X		X		
miR-17-5p	478447			X	X	
miR-19a-3p	479228	X		X		
miR-19b-3p	478264	X		X		
miR-20a-5p	478586	X		X		
miR-21-5p	477975	X		X		
miR-22-3p	477985	X		X		
miR-23a-3p	478532	X	X	X		
miR-23b-3p	478602			X		
miR-24-3p	477992	X		X	X	
miR-25-3p	477994	X		X		
miR-26a-5p	477995	X		X		
miR-26b-5p	478418			X		
miR-27a-3p	478384	X		X		
miR-27b-3p	478270	X		X		
miR-27b-5p	478789			X		
miR-28-5p	478000			X		
miR-29a-3p	478587		X	X		
miR-29c-5p	478005			X		
miR-30a-3p	478273	X		X		
miR-30c-1-3p	479412			X		
miR-30c-2-3p	479401			X		
miR-30c-5p	478008			X		
miR-30d-5p	478606			X		
miR-30e-3p	478388			X		
miR-31-5p	478015	X		X		
miR-32-5p	478026			X		
miR-34a-3p	478047			X		
miR-34a-5p	478048	X		X		
miR-34b-5p	478050			X		
miR-34b-3p	478049			X		
miR-34c-3p	478051			X		
miR-34c-5p	478052			X		

miR-92a-3p	477827			X	
miR-92b-3p	477823			X	
miR-93-5p	478210	X		X	
miR-99a-3p	479224			X	
miR-99b-5p	478343			X	
miR-100-3p	478619			X	
miR-100-5p	478224			X	
miR-101-3p	477863		X	X	
miR-103a-3p	478253			X	X
miR-103a-2-5p	477864			X	
miR-106b-3p	477866			X	
miR-106b-5p	478412	X		X	
miR-107	478254		X	X	
miR-122-5p	477855			X	
miR-124-3p	477879		X	X	
miR-125a-5p	477884		X	X	
miR-125a-3p	477883			X	
miR-125b-5p	477885	X	X	X	
miR-126-5p	477888			X	
miR-127-3p	477889			X	
miR-128-3p	477892	X	X	X	
miR-129-2-3p	478544			X	
miR-130a-3p	477851			X	
miR-132-3p	477900	X	X	X	
miR-133a-3p	478511			X	
miR-133b	480871			X	
miR-135a-5p	478581		X	X	
miR-137	477904		X	X	
miR-142-3p	477910	X		X	
miR-142-5p	477911			X	
miR-143-3p	477912			X	
miR-144-3p	477913	X		X	
miR-145-3p	477915			X	
miR-145-5p	477916	X		X	
miR-146a-5p	478399	X		X	
miR-146b-5p	478513	X		X	
miR-148a-3p	477814	X		X	
miR-148b-3p	477824			X	
miR-150-5p	477918	X		X	
miR-151a-3p	477919			X	
miR-151a-5p	478505			X	
miR-153-3p	477922		X	X	
miR-155-5p	477927	X		X	
miR-181a-5p	477857	X	X	X	
miR-181b-5p	478583			X	
miR-181c-5p	477934	X		X	
miR-181d-5p	479517			X	
miR-183-3p	477936		X	X	
miR-185-5p	477939			X	
miR-186-5p	477940	X		X	X
miR-190a-5p	478358		X	X	
miR-191-3p	477951			X	
miR-191-5p	477952			X	X
miR-193a-5p	477954	X		X	
miR-194-5p	477956			X	
miR-195-5p	477957			X	
miR-196a-5p	478230			X	

miR-199a-3p	477961			X
miR-199a-5p	478231			X
miR-200c-3p	478351			X
miR-203a-3p	478316			X
miR-204-5p	478491			X
miR-205-5p	477967			X
miR-206	477968			X
miR-210-3p	477970			X
miR-211-5p	478507			X
miR-216a-5p	477976			X
miR-218-5p	477977			X
miR-219a-5p	477980		X	X
miR-221-3p	477981			X
miR-222-3p	477982			X
miR-223-3p	477983	X		X
miR-302b-3p	478591			X
miR-302d-3p	478237			X
miR-320a	478594	X		X
miR-320b	478588			X
miR-323a-3p	477853		X	X
miR-325	478025			X
miR-326	478027	X		X
miR-328-3p	478028			X
miR-335-5p	478324		X	X
miR-338-3p	478037		X	X
miR-339-5p	478040			X
miR-342-3p	478043			X
miR-361-3p	478055			X
miR-361-5p	478056			X
miR-363-3p	478060			X
miR-369-3p	478067			X
miR-369-5p	478068			X
miR-373-3p	478363			X
miR-374b-5p	478389			X
miR-375	478074			X
miR-376a-3p	478240			X
miR-376c-3p	478459	X		X
miR-378a-3p	478349			X
miR-378a-5p	478076			X
miR-383-5p	478079		X	X
miR-410-3p	478085			X
miR-411-5p	478086		X	X
miR-412-3p	478087			X
miR-423-5p	478090			X
miR-424-5p	478092			X
miR-425-5p	478094			X
miR-448	478105			X
miR-449a	478561			X
miR-449b-5p	479528			X
miR-450b-3p	478913			X
miR-450b-5p	478914			X
miR-451a	478107	X		X
miR-452-3p	478917			X
miR-452-5p	478109			X
miR-454-3p	478329	X		X
miR-455-3p	478112			X
miR-483-3p	478122			X

miR-483-5p	478432			X
miR-484	478308	X		X
miR-486-5p	478128			X
miR-487a-3p	477826			X
miR-489-3p	478130			X
miR-490-3p	478131			X
miR-497-5p	478138	X		X
miR-501-3p	478350			X
miR-502-3p	478348			X
miR-505-3p	478145			X
miR-513a-5p	479483			X
miR-515-3p	478976			X
miR-516b-5p	478979			X
miR-518d-3p	479393			X
miR-518e-3p	479408			X
miR-518f-3p	478984			X
miR-520h	479499			X
miR-523-3p	478994			X
miR-524-3p	479338			X
miR-525-3p	478995			X
miR-532-3p	478336			X
miR-532-5p	478151			X
miR-548d-5p	480870			X
miR-548e-3p	478362			X
miR-548k	479374			X
miR-548n	479024			X
miR-551a	478158			X
miR-570-3p	479053			X
miR-576-3p	478164			X
miR-583	479065			X
miR-593-5p	479077			X
miR-615-3p	478175			X
miR-628-3p	478181			X
miR-633	479115	X		X
miR-642a-5p	479121			X
miR-645	478188			X
miR-652-3p	478189			X
miR-653-3p	479134			X
miR-656-3p	479137			X
miR-660-5p	478192	X		X
miR-664a-3p	478193	X		X
miR-770-5p	479178			X
miR-876-3p	479186			X
miR-885-5p	478207			X
miR-937-3p	479212			X
miR-939-5p	478245	X		X
miR-1247-5p	477882			X
miR-1249-3p	478654			X
miR-1260a	478476			X
miR-1264	478670			X
miR-1292-5p	478691			X
miR-1298-5p	479452			X
miR-1911-5p	479583		X	X

Table 2. SSS scores for CSF samples of each studied group.

miRNA name	OND SSS Score	PPMS SSS Score	RRMS SSS Score	SAS SSS Score
let-7a-5p	2.072 (21)	4.079 (56)	2.616 (36)	1.960 (5)
let-7b-5p	2.203 (31)	3.175 (41)	2.101 (7)	2.582 (35)
miR-101-3p	1.676 (5)	2.894 (29)	2.199 (14)	2.168 (15)
miR-124-3p	1.624 (1)	2.700 (15)	2.536 (33)	2.468 (32)
miR-125a-5p	2.193 (30)	2.265 (1)	2.097 (6)	2.028 (9)
miR-143-3p	2.408 (40)	2.673 (12)	2.250 (16)	1.834 (3)
miR-150-5p	1.943 (13)	2.684 (13)	2.187 (13)	1.986 (7)
miR-151a-3p	2.470 (43)	2.516 (9)	2.365 (22)	2.350 (23)
miR-15a-5p	1.926 (11)	2.438 (6)	2.481 (27)	2.702 (39)
miR-181a-5p	2.086 (23)	2.802 (20)	2.117 (8)	2.380 (24)
miR-195-5p	2.525 (47)	9.053 (66)	2.117 (9)	1.791 (2)
miR-21-5p	1.916 (10)	2.772 (18)	2.119 (10)	1.909 (4)
miR-22-3p	1.668 (4)	2.506 (8)	2.457 (25)	2.286 (20)
miR-221-3p	2.075 (22)	2.836 (24)	2.048 (3)	2.393 (26)
miR-23a-3p	2.139 (27)	2.325 (2)	2.371 (23)	1.787 (1)
miR-26b-5p	1.661 (3)	2.353 (3)	2.023 (2)	1.980 (6)
miR-27a-3p	1.886 (8)	2.780 (19)	2.074 (4)	2.136 (12)
miR-335-5p	1.643 (2)	2.446 (7)	2.155 (11)	2.008 (8)
miR-449b-5p	2.023 (17)	5.650 (62)	3.493 (62)	2.060 (10)
miR-652-3p	1.684 (6)	2.657 (10)	2.494 (28)	2.604 (37)
miR-660-5p	1.902 (9)	2.358 (4)	2.754 (45)	2.279 (19)
miR-9-5p	2.164 (28)	2.880 (28)	2.081 (5)	2.336 (21)
miR-92b-3p	1.759 (7)	2.386 (5)	1.992 (1)	2.111 (11)

SSS miRNA stability scores are represented for each studied group of patients and its ranked position from the total set of miRNAs is in brackets. OND: other neurological diseases; PPMS: primary progressive multiple sclerosis; RRMS: relapsing-remitting multiple sclerosis; SAS: spinal anesthesia subjects; SSS: summarized stability score.

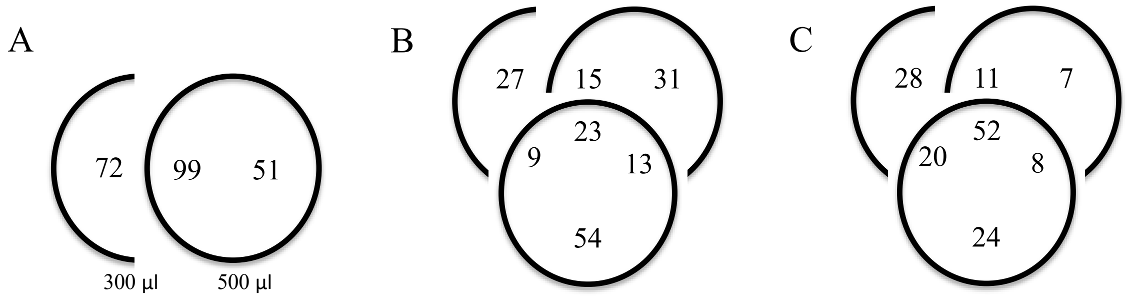

Figure S1. Venn diagram plot the number of detected miRNAs in different conditions. A) Left circle corresponds to samples of 300 µl of CSF, right circle corresponds to samples of 500µl of CSF. B) Each circle corresponds to a specific sample of CSF of 300 µl of starting volume. C) Each circle corresponds to a specific sample of CSF of 500 µl of starting volume.

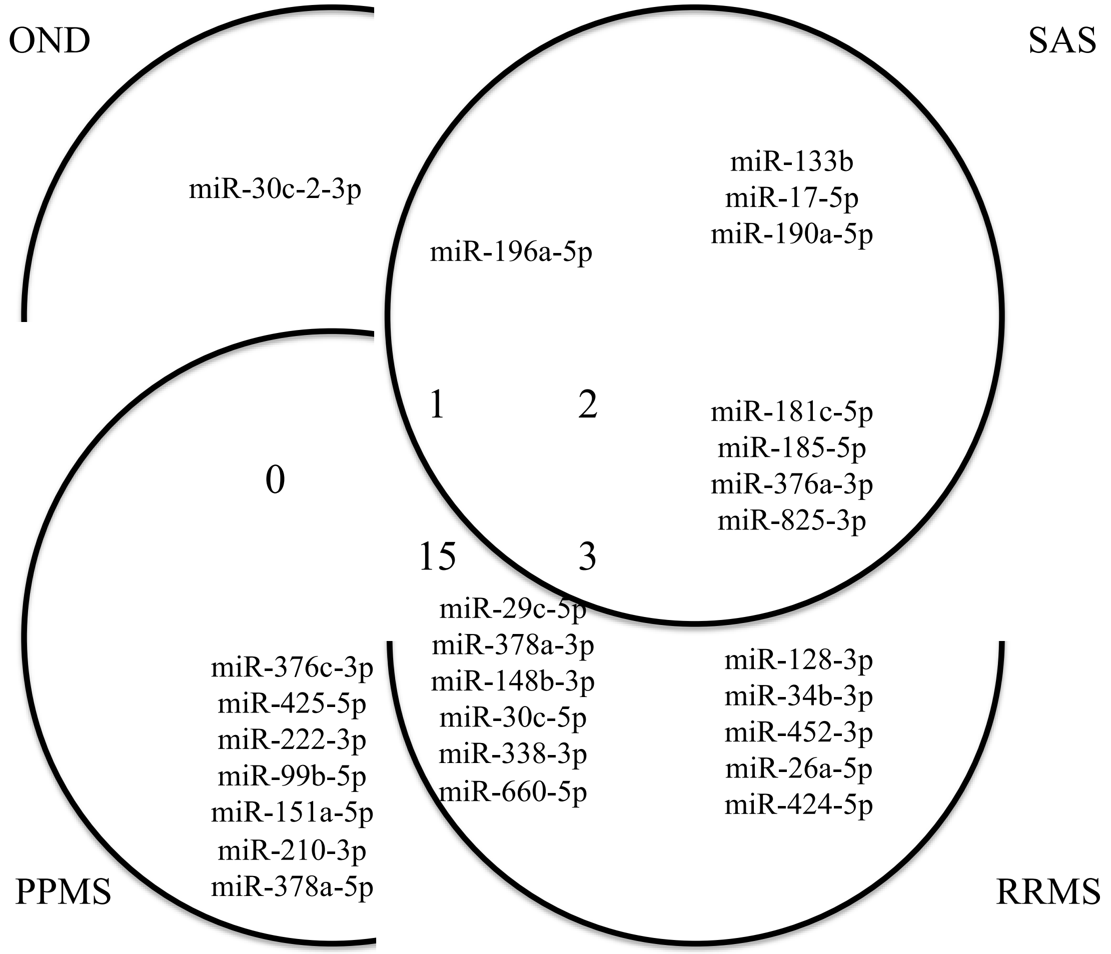

Figure S2. Venn diagram plot the number of miRNAs detected in at least 70% of samples in each group of patients. Each circle corresponds to one specific groups of samples. OND: other neurological diseases; PPMS: primary progressive multiple sclerosis; RRMS: relapsing-remitting multiple sclerosis; SAS: spinal anesthesia subjects.

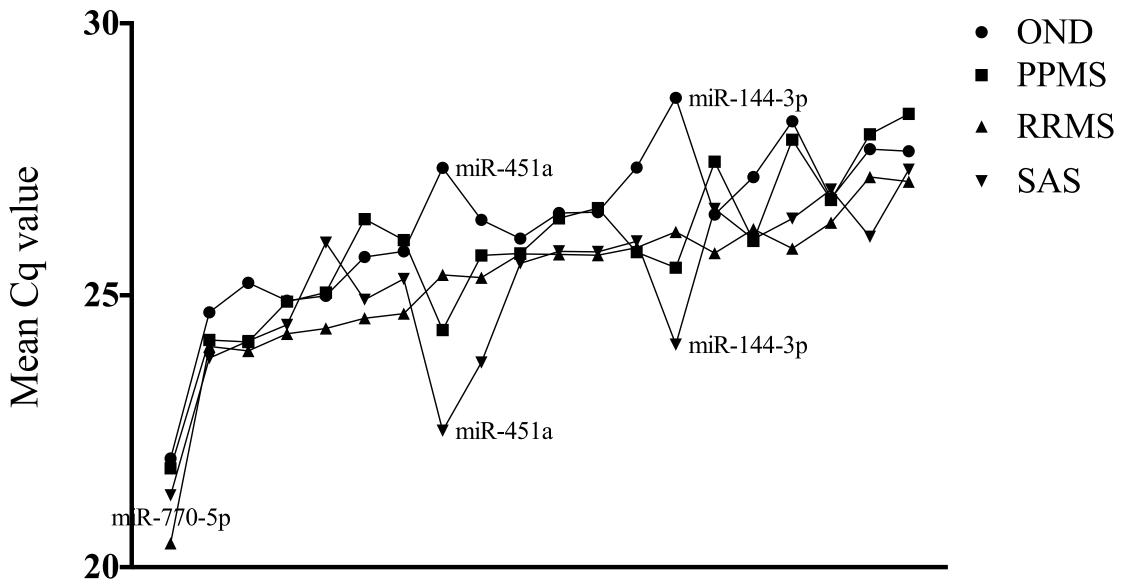

Figure S3. Mean Cq value of the most abundant miRNAs in CSF in each specific group of studied samples. Circle corresponds to OND; square corresponds to PPMS; triangle corresponds to RRMS and; inverted triangle corresponds to SAS individuals.
